# From pattern separation to mood regulation: multiple roles for developmental signals in the adult dentate gyrus

**DOI:** 10.3389/fncel.2013.00096

**Published:** 2013-06-26

**Authors:** Marlena Wosiski-Kuhn, Alexis M. Stranahan

**Affiliations:** Physiology Department, Georgia Health Sciences UniversityAugusta, GA, USA

## Overview of adult hippocampal neurogenesis

The dentate gyrus represents a unique system for the study of interactions between neuronal development and experience. While this hippocampal subfield has been extensively investigated in relationship to the ongoing process of adult neurogenesis, the mnemonic contributions of mature granule neurons have remained enigmatic. Much of the rationale for focusing on newly generated neurons, as opposed to mature granule cells, rests on the greater excitability of immature neurons, which positions them to make a unique contribution to memory processing. On the other hand, the assertion that mature granule cells are either inactive, or very finely tuned to specific spatial configurations, is derived from electrophysiological recording experiments and measures of immediate early gene expression conducted under conditions that are not likely to evoke significant psychological or physiological stress. In this Opinion piece, we present the argument that mature dentate granule neurons are synaptically silent under low-stress conditions, but are recruited in far greater numbers under emotionally salient conditions due to their combined input from the amygdala and entorhinal cortex. Functional activation of the far more numerous mature granule cell population could potentially serve as an emotional tag linking spatial context with stressful experiences. The underlying mechanism for emotional tagging of spatial contexts likely involves signaling cascades implicated in neuronal development, such as the reelin signaling pathway. Alterations in reelin signaling among mature granule neurons could therefore determine their ability to disambiguate stressful and non-stressful contexts.

Adult born neurons account for up to 10% of the entire granule cell population (Imayoshi et al., 2008), and at the point of senescence, nearly forty percent of granule neurons will have been born in adulthood (Snyder and Cameron, 2012). The functional significance of dentate neurogenesis is thought to arise from the distinct synaptic properties of newly generated neurons, with the assumption that increased excitability among new neurons favors their recruitment over the mature dentate granule neuron population. A number of studies have hypothesized mature granule neurons may in fact be functionally silent (Aimone et al., 2010; Alme et al., 2010), but an alternative interpretation can be generated by comparing across studies that evaluated the recruitment and functional significance of dentate gyrus granule cells under low- or high-stress conditions. Although the distributed innervation of dentate granule neurons favors sparse coding, with approximately three percent of granule cells exhibiting firing during exploration of an environment (Chawla et al., 2005; Leutgeb et al., 2007; Treves et al., 2008), these recordings were conducted under low-stress conditions that may not recruit as many mature dentate granule cells. Sparse activation of functionally mature dentate granule cells is sufficient for recall of contextual fear memory (Liu et al., 2012), suggesting that although the proportion of mature granule cells recruited by an experience may be relatively small, the selective recruitment of specific ensembles is involved in the behavioral expression of memory for stressful contexts. Given that the dentate gyrus receives extensive innervation from the amygdala (Wheal and Miller, 1980; Bergado et al., 2007)), mature granule cells may be functionally poised for activation only under conditions of high emotional salience.

## Physiological and properties of adult-born neurons in memory encoding

**Figure 1:**
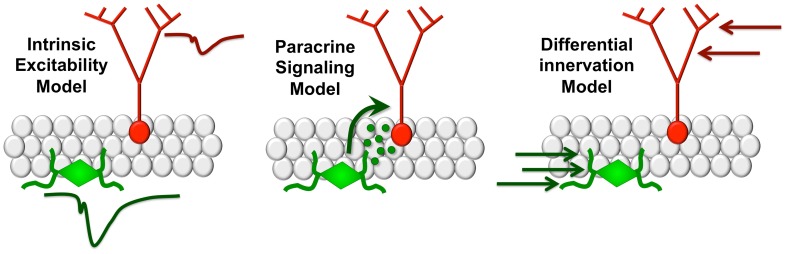
**Modeling functional interactions between new and mature dentate gyrus granule cells.** The intrinsic excitability model **(left panel)** suggests that differences in firing thresholds between new and mature neurons favor recruitment of the immature neuronal population. In the paracrine signaling model **(center)**, new neurons release signaling molecules that influence plasticity among mature granule cells. Under the differential innervation model **(right)**, new neurons respond differently due to distinct patterns of anatomical innervation by local circuits.

Adult-born granule cells have a lower threshold for long-term potentiation (LTP), and exhibit enhanced synaptic plasticity relative to mature granule neurons (Schmidt-Hieber et al., 2004; Ge et al., 2007a; Mongiat et al., 2009). New neurons in the adult brain recapitulate developmental transitions from excitatory responses to GABA to inhibitory responses during the first three days post-mitosis (Ge et al., 2007b). At later time points following this transition, new neurons continue to exhibit enhanced LTP and reduced threshold for LTP induction (Ge et al., 2007a). At four weeks of age, newly generated granule cells are functionally integrated into the perforant path circuitry, with afferents along the mossy fiber pathway to CA3 (Toni et al., 2007), but remain distinguishable based on their increased morphological plasticity, particularly among dendritic spines (Zhao et al., 2006). Each of these properties has prompted extensive speculation regarding a selective role for adult-generated neurons in learning, but the possibility that new neurons release signaling molecules that influence plasticity among mature neurons has yet to be adequately addressed. It is possible that adult neurogenesis influences recruitment and plasticity among mature neurons, thereby regulating dentate gyrus contributions to pattern separation and memory (Figure 1).

Ablation of adult hippocampal neurogenesis impairs pattern separation, defined as the ability to disambiguate similar spatial contexts (Deng et al., 2010; Tronel et al., 2012). X-ray irradiation of adult born neurons impairs discrimination between two similar contexts during contextual fear conditioning (Sahay et al., 2011) but the specific developmental window for this effect remains obscure due to methodological variability with respect to the interval between irradiation and memory assessment. More recent studies (Arruda-Carvalho et al., 2011) used a novel approach to identify and silence adult generated neurons both before and after training and examine the subsequent effects on memory recall. Silencing of adult-generated neurons before learning did not prevent the formation of new contextual fear or water maze memories, but ablation of new neurons after learning degraded existing contextual and spatial memories. An essential role for adult-generated neurons after, but not before, training on a memory task is consistent with a potential paracrine signaling mechanism allowing new neurons to influence the larger population of mature neurons to facilitate encoding (Figure 1).

The possibility of a distinct functional contribution for adult born granule cells relative to those born during development could be explained by anatomy, rather than by intrinsic functional properties or a paracrine signaling mechanism. Dendritic architecture varies with position in the granule cell layer (Desmond and Levy, 1982; Claiborne et al., 1990; Redila and Christie, 2006) and granule cells born in adulthood are deeper in the granule cell layer than those born in development. Therefore the functional contributions of new neurons may arise from the properties of the subgranular zone microenvironment rather than their excitability or a potential signaling mechanism impacting the larger population of mature dentate granule cells. Vascular innervation of the subgranular zone is substantially more dense than the granule cell layer or molecular layer of the dentate gyrus (Monje et al., 2003), opening the possibility that new neurons might exhibit distinct patterns of neurovascular coupling based on their greater spatial proximity to blood vessels. Understanding how neuronal neighborhoods influence the functional contributions of specific subpopulations of cells is an important and understudied area, both in the dentate gyrus and other hippocampal subfields.

Adult born neurons are first incorporated into local circuits before receiving long range input (Deshpande et al., 2013). In normal mice, incorporation of newborn granule cells into the trisynaptic circuit only occurs once they have reached functional maturity on the cellular level. Interestingly, at this timepoint new neurons also begin to be innervated by the subiculum, which is implicated in the stress response (Herman and Mueller, 2006). Effective termination of the corticosteroid response to stress requires adult-born neurons, but the extent to which subicular innervation of new neurons contributes to this functional role remains unclear (Snyder et al., 2011). Given the extensive speculation surrounding alterations in adult neurogenesis following stress, and in animal models of depressive-like behavior, it is essential to understand the functional role of new and mature dentate granule neurons under conditions of high and low emotional valence.

Fear conditioning tasks are dependent on both basolateral amygdala (BLA) and hippocampus, as seen in cooperative induction of MAPK/ERK signaling components and immediate early genes in the hippocampus and amygdala following contextual fear conditioning and retrieval (Maren, 1996; Maren et al., 1996; Goosens and Maren, 2001; Roozendaal et al., 2009). Therefore, the specific contributions of new neurons to fear conditioning tasks could arise from differential innervation of new and mature neurons by the amygdala. Immature neurons, unlike the mature granule cell population, are primarily activated in the ventral blade of the dentate during water maze learning, opening the possibility of a specific role in regulating the response to stress and emotional learning (Snyder et al., 2009). However, specific information on differential innervation of the dorsal and ventral dentate blades by the amygdala (or any other region) remains scarce. Cholinergic inputs from the BLA to the hippocampus go through the medial septum, while glutmatergic inputs arrive via the entorhinal cortex (Wheal and Miller, 1980; Bergado et al., 2007). Both sets of inputs are poised to modulate adult neurogenesis, given that acetylcholine and glutamate increase proliferation of adult neural precursor cells (Ge et al., 2007b). In a recent study (Kirby et al., 2012), immediate early gene expression was evoked in new neurons by fear memory training, but not by exposure to a new environment. BLA lesions led to reductions in adult neurogenesis and impairment of immediate early gene expression in the remaining new neuronal population (Kirby et al., 2012). These observations underscore the importance of amygdalar inputs to the dentate gyrus in the regulation of both memory and neuronal recruitment under high-stress conditions.

## Developmental molecules in neurogenesis and neuronal maturation

The glycoprotein reelin has been heavily implicated in a variety of stress-related neuropsychiatric conditions and is widely expressed among interneurons in the adult vertebrate brain (Pérez-García et al., 2001). Excitatory layer II entorhinal cortical neurons also express reelin, both in the cell body, and in the axons that form the perforant path projection to the dentate gyrus (Stranahan et al., 2009). While the dentate gyrus is innervated by reelinergic afferents from the entorhinal cortex, retroviral overexpression or inactivation of reelin specifically in new neurons supports a cell autonomous role for reelin glycoprotein in adult neurogenesis (Pramatarova et al., 2008). Reelin overexpression accelerated dendritic maturation, while inactivation of reelin in adult caused aberrant migration, decreased dendrite development, and elicited formation of ectopic dendrites in the hilus. Interestingly, deletion of reelin during adult neurogenesis increases the number of glia at the expense of neurons compared to wild types, indicating that reelin controls progenitor cell fate not only in development but also in adulthood.

Reelin facilitates adult hippocampal neurogenesis by promoting the correct migration and orientation of newly born granule cells, but may also play a role in controlling synaptic plasticity among the mature granule cell population. A number of developmentally regulated signaling molecules such as cyclin-dependent kinase 5, Notch, and WNT have been implicated in the regulation of adult dentate granule cell neurogenesis, and many of these signaling pathways overlap with reelin. The challenge in understanding how these signaling pathways influence dentate circuitry is directly related to the developmental heterogeneity of dentate gyrus granule cells; it remains uncertain whether reelin, or any other neurodevelopmental signal, impacts dentate synaptic plasticity by influencing new neurons, mature neurons, or communication between new and mature neurons, and the likely outcome is that each of these scenarios will be upheld in different contexts. The extent to which a repeated experience evokes firing among distinct or overlapping ensembles of neurons at various stages of maturation may determine the fidelity and accuracy of contextual discrimination, in part by recruiting signaling pathways associated with neural development.

## Conclusion

While the dentate circuitry is most studied with respect to adult neurogenesis, the functional role of the mature granule cell population remains mysterious. Experiments addressing the contributions of the far more numerous populations of mature granule neurons would benefit from the inclusion of high- and low-stress conditions, in order to activate amygdalar projections to the dentate gyrus. Although there has been significant speculation that mature granule neurons are essentially “retired,” the possibility remains that they may have simply changed “careers,” by refining their responses based on emotional arousal in addition to cognitive demands.
